# Surgery

**Published:** 1897-03

**Authors:** W. M. Barclay


### SURGERY.

Among the rarer diseases that may have to be reckoned with at any time **acute pancreatitis** is beginning to take its place in abdominal surgery. It is eight years since Dr. Fitz wrote his exhaustive paper,[1] in which he gathered into a focus all the scattered knowledge on the subject, and enriched it with fresh cases from his own practice, and by the light which his careful analysis threw on the symptomatology and pathology of the affection; and it still remains the standard authority. The publication of cases since has only served in the main to justify his conclusions, although a few minor corrections or amplifications have necessarily resulted from the more accurate observation and the fuller recognition of the condition which his own work inaugurated. Dr. Fitz's paper is based on the consideration of seventy cases, all of which he has carefully tabulated. It will make the subject clearer if his general conclusions are first set down, reserving his classification of the cases, and any more recent observations, for consideration afterwards. He comes to the conclusion that acute inflammation of the pancreas is a well-characterized disease, of much greater frequency than is generally supposed; that it is important, as presenting usually a serious complication of a relatively simple affection, viz. gastro-duodenitis; as a cause of peritonitis that may easily be overlooked; and because it has been repeatedly confounded with acute intestinal obstruction. The common symptoms of this condition are "sudden, severe, often intense, epigastric pain, without obvious cause, in most cases followed by nausea, vomiting, sensitiveness, and tympanitic swelling of the epigastrium. There is prostration, often extreme, frequent collapse, low fever, and a feeble pulse. Obstinate constipation for several days is the rule, but diarrhœa sometimes occurs. If the case does not end fatally in the course of a few days, recovery is possible, or a recurrence of the symptoms in a milder form takes place, and the characteristics of a sub-acute peritonitis are developed." These symptoms he considers to be essentially those of a peritonitis beginning in the epigastrium, and occurring suddenly, during ordinary health, without obvious cause, and the differential diagnosis to lie between (1) an irritant poison; (2) a perforation in the digestive or biliary tract; and (3) acute intestinal obstruction. 1. The first must be excluded by the history and an examination of the vomit. 2. Perforating ulcer of the stomach or duodenum should be negatived by the absence of pain after eating, of hemorrhages from the digestive canal, and of cachexia; acute perforation of the transverse colon is rare, and the resulting peritonitis progresses more rapidly, and is likely to be general; and perforation from gall-stones is usually preceded by attacks

[1] *Med. Rec.*, 1889, xxxv. 197, 225, 253.

of colic and jaundice, while the seat of the pain is more in the region of the gall-bladder. 3. The diagnosis from acute intestinal obstruction is often most difficult. Dr. Fitz suggests that the patency and capacity of the large intestine may be determined by an injection, and reminds us of the rarity of an obstructed small intestine in the epigastric region; and the immediate presence of a localised tenderness and the usual absence of a conspicuous general tympany, or limited distension of intestinal coils, are referred to as aids in differentiation. It will be obvious from a consideration of the above how difficult the diagnosis must be in many cases, and it may well prove insurmountable in some.[1]

Dr. Fitz classifies cases of acute pancreatitis into (1) hemorrhagic; (2) suppurative; and (3) gangrenous. These varieties have more or less distinguishing features, and the mode of onset and the course of the disease present well-marked peculiarities.

1. **Hemorrhagic pancreatitis.**—All the cases occurred in adults, between the ages of 25 and 60,[2] and in nearly half the number there was a history of previous digestive troubles. The pain was either constant or paroxysmal, usually beginning in the upper abdomen, but in a fifth of the cases subsequently becoming general; vomiting might be constant, repeated, or only occasional; constipation was common; and symptoms of collapse almost invariably supervened, with death in from two to six days. The pancreas was found enlarged, either uniformly or at one end; its consistence was dense, or friable; and it was red on the surface, while internally the blood formed a patchy or uniform extravasation, in all shades of red and purple to black. The section might be further altered by bands and spots of yellowish fat, or opaque white specks, spots, or streaks of "fat-necrosis." The hemorrhage often extended beyond the gland into the parapancreatic tissue, mesentery, mesocolon, and omentum, or even to the outer border of the left kidney, or downwards behind the descending colon almost to the level of Poupart's ligament. There was usually no peritonitis. The pathological condition found was that of cellular or fibrinocellular infiltration, especially affecting the interlobular tissue, with extensive hemorrhage; and "coagulation-necrosis" in many of the lobules.

In connection with this variety of acute pancreatitis, it may be well to refer to an analogous (though apparently distinct) condition, that of **hemorrhage into the pancreas,**[3] which comes on without known cause, and is so rapidly fatal (half-an-hour to thirty-six hours) as to be well called "apoplectiform." The patients were adults, usually over forty, who were seized with a sudden acute, or trifling, pain, with nausea and occasionally a little vomiting, and either constipation or diarrhœa, but in

[1] See a case reported in *Lancet*, 1897, i. 36.

[2] Recently a case has been published by Dr. McPhedran in an infant of nine months old. *Canad. Pract.*, 1896, xxi. 650.

[3] Fitz, *loc. cit.*

whom fatal collapse invariably supervened. The hemorrhage took place into and around the gland as in acute pancreatitis, but with no evidence of inflammation. Dr. Hawkins,[1] in reporting such a case, with microscopical sections of the gland, suggests that some cases at least of acute pancreatitis may arise from a primary simple hemorrhage, inflammation taking place secondarily around the necrosed parts. If the patient survive the first shock, and the destruction of gland-tissue be not too great, the necrosed parts, he thinks, should be capable of absorption, and he refers to a case quoted by Osler,[2] where laparotomy was performed for supposed intestinal obstruction, in which only injected and distended coils were found, without any obstruction, but presenting in the region of the pancreas, and at the root of the mesentery, a dense, thick, indurated mass, with areas of "fat-necrosis" in the mesentery and omentum. The patient recovered. He thinks pancreatitis is very likely due to an invasion by micro-organisms along the ducts. The severe and fatal collapse in these cases, and in acute pancreatitis, has been attributed by Zenker[3] and Cayley[4] to interference with the functions of the great sympathetic ganglia and consequent vaso-motor paralysis of the mesenteric vessels, together with cardiac inhibition. The latter, therefore, suggests that, in addition to the ordinary means of combating shock, suprarenal extract might do good from its reputed efficacy in controlling vaso-motor paralysis and stimulating the heart. Gentle abdominal pressure, if bearable, might be adopted also.

2. **Suppurative pancreatitis.** — The patients were adults, between 20 and 74, and in a quarter of the cases previous attacks of "indigestion" were noted. An important feature was its tendency to become chronic. The cases were accordingly subdivided into acute, subacute, and chronic. The acute cases usually began suddenly with urgent symptoms of the general type; the bowels were generally constipated, but sometimes diarrhœa occurred in the first twenty-four hours, and was not infrequent at a later stage; fever often developed about the third day, with abdominal swelling, which at first was epigastric and then general. Rarely the case ended fatally in a week. Much more commonly the symptoms persisted for three or four weeks, and then the patient died exhausted: or chills, with an atypical febrile temperature, might succeed, the liver and spleen become manifestly enlarged, and the patient die in six or seven weeks of blood-poisoning. In more subacute cases there might be an interval of several weeks of comparative comfort, only to be followed, however, by marked prostration and death: or diarrhœa might become a conspicuous symptom in the third or fourth week, then subside, and again recur with a fatal issue: or the course might be still less acute, the early symptoms being

[1] *Lancet*, 1893, ii. 358. [2] *The Principles and Practice of Medicine*, 1892, p. 459.
[3] Quoted by Fitz, *loc. cit.* [4] *Brit. M.J.*, 1896, ii. 1.

progressive weakness and emaciation, with perhaps slight jaundice and diarrhœa; there might be neither fever nor pain, or obscure symptoms of peritonitis might declare themselves; finally, anasarca and ascites led to death in about five months. Lastly, there were the chronic cases, which varied in duration from five to eleven months. Here there was the same insidious onset as noticed above; vomiting might be frequent, and distension of the stomach after meals, or epigastric pain, be complained of for some time; the stools were consistent, colourless, and very fœtid, and later might be mixed with blood; a swollen, tympanitic epigastrium was the rule. According to Dr. Fitz, it was only very rarely that a circumscribed tumour, or a circumscribed resistent spot above and to the left of the navel, was to be felt. Dr. J. W. Elliot,[1] however, reported three cases of suppurative pancreatitis in his own practice, in two of which a well-defined tumour was present; and he quotes three similar instances from Körte,[2] another of Musser's,[3] and four cases cited by Körte in his paper—a total of ten cases. From these it would appear that such tumours may be found, (1) as an epigastric swelling behind the stomach, due to pus in the omental sac; (2) running from the epigastrium to the spleen, just below the ribs, due to an enlarged pancreas or a para-pancreatic abscess; (3) as a swelling in the loin, or more rarely on the right side below the liver, from the burrowing of a retro-peritoneal pancreatic abscess. It may be difficult or impossible to feel such tumours, if the abdominal wall is very fat (and this seems often to be the case) or if the abdomen is very tympanitic and painful; they are deeply placed, and are often tympanitic on percussion from over-lying bowel. Careful palpation is the best way of discovering their presence.

The condition of the pancreas varied in these cases: it might be enlarged and studded with hundreds of small abscesses; a simple abscess might be found surrounded with adhesions; several small abscesses in an enlarged and firm gland; diffuse abscess following the course of the ducts and opening into the cavity of the omentum; or the entire pancreas might be found converted into a trabeculated cavity filled with creamy pus and cheesy masses.

3. **Gangrenous pancreatitis.**—The ages varied from 22 to 63; in half the cases the patients had been subject to repeated attacks of digestive disturbance. In four-fifths of the cases it began suddenly, without definite cause; the pain was generally severe, and might be referred to the stomach, left hypochondrium, navel, mid-abdomen, or left loin and back; vomiting was very common; the condition of the bowels varied, in some cases constipation being pronounced, while in others frequent bilious stools occurred; fever of a low type often developed after a few days, but might be absent throughout; swelling of

[1] *Boston M. & S. J.*, 1895, cxxxii. 352.
[2] *Arch. f. klin. Chir.*, 1894, xlviii. 721. [3] *Am. J. M. Sc.*, 1886, xci. 449.

the epigastrium, or left half of the abdomen, occurred in half the cases, usually late in the course of the disease, producing a fluctuating tumour which was generally tympanitic, and was either slightly marked or of enormous size; suggestions of peritonitis were frequent, and collapse was very likely to supervene; and death took place in from four days to eight weeks.

The pancreatic changes varied according to the acuteness of the case apparently, or from other circumstances: the gland might be enlarged, dark and mottled, and shreddy in parts; or form a slaty, stinking slough in its entirety; it sometimes lay bathed in dirty green fluid, or in a cavity filled with ichorous greasy pus and detritus, the gland being firm, or flabby and friable, and barely, if at all, attached to its containing cavity.

The **causation** of acute pancreatitis is obscure. Fitz believes that it usually originates by the extension of a gastro-duodenal inflammation along the ducts; but that it may also be induced by the occurrence of hemorrhage into the gland. In either case the inflammation is probably microbic.

The **prognosis** is exceedingly grave, and almost hopeless in the majority of cases. The apoplectiform hemorrhage seems to be invariably fatal, and the same may almost be said for the hemorrhagic variety of acute pancreatitis: Osler's case of recovery must, however, be remembered, the more so as it is difficult to attribute the happy result to the effect of the laparotomy. In the suppurative and gangrenous varieties the chances of recovery are a little better, and it is here that surgical interference seems most hopeful of some good result. Fitz could record no case of recovery from suppurative pancreatitis in his table, but two cases have since been published where operation was successful—one of Körte's[1] cases and one recorded by Thayer.[2]

Suppurative pancreatitis may result in evacuation of the abscess into the stomach or intestines, directly, or indirectly through the omental sac; but recovery by such a termination must be very rare. Two cases of spontaneous evacuation of a necrosed pancreas have been reported, with complete recovery.[3]

*Treatment.*—All authorities seem to agree that the acute stage is unfavourable for operation, when, that is, the patient is in a state of collapse. After reaction has set in (if it does), in some cases the surgeon may be able to interfere with advantage. Elliot[4] thinks it conceivable that diseased portions of the pancreas might be excised successfully, and it seems not unreasonable to hope that a sloughing gland might be occasionally removed with a good result. Elliot bases his hopes in surgical interference on the following facts: (1) The presence of scars in the pancreas at necropsies, showing that a limited pancreatitis may recover; and one of the cases of evacuation of a sloughed gland *per rectum*, referred to above. (2) Experiments on dogs,

[1] *Loc. cit.* [2] *Am. J. M. Sc.*, 1895, cx. 396. [3] Fitz, *loc. cit.* [4] *Loc. cit.*

which show that part of the pancreas may be removed without impairment of health. In man the successful removal of the tail of the pancreas has been reported by Kleberg.[1] (3) Körte's successful operation for suppurative pancreatitis. He directs that, if pus has collected in the omental bursa, the incision should be made in the middle line from the xiphoid cartilage downwards, and the bursa opened by tearing through the omentum between the stomach and transverse colon. The omentum can be stitched to the abdominal wall before opening, thus shutting off the peritoneal cavity. If the abscess-cavity extends along the pancreas, a counter-opening should be made in the lumbar region just below the twelfth rib. When the pus is evidently retro-peritoneal, and burrowing in the lumbar region or in the mesocolon, the opening should be made in the back, as for extirpation of the kidney, and then with a blunt dissector the pus-cavity should be searched for without opening the peritoneum.

The condition of "fat-necrosis" in the peritoneum around and in the neighbourhood of the pancreas has been noted in a large majority of all the cases. Its pathology and significance are obscure, and it has been observed where the pancreas was perfectly healthy; yet its association with these cases can scarcely be entirely fortuitous, and in any case its presence may be practically important. It occurs in the form of opaque white spots or masses, from the size of a pin's head to that of half a split-pea, or in rarer instances much larger (as large as a hen's egg[2]) which are either flat, or raised above the surface. In the latter case they may strongly suggest miliary tubercles, or malignant nodules, according to their size. Such a mistake might lead to the abandonment of an otherwise hopeful operation, and cause a grave error. On the other hand, if duly recognised, their presence would be a suggestive clue in an obscure exploratory laparotomy.

W. M. Barclay.

[1] *Arch. f. klin. Chir.*, 1868, ix. 525. [2] Fitz, *loc. cit.*

